# Route and Type of Formulation Administered Influences the Absorption and Disposition of Vitamin B_12_ Levels in Serum

**DOI:** 10.3390/jfb9010012

**Published:** 2018-01-21

**Authors:** Luis Vitetta, Joyce Zhou, Rachel Manuel, Serena Dal Forno, Sean Hall, David Rutolo

**Affiliations:** 1 Sydney Medical School, The University of Sydney, Sydney 2006, Australia; 2 Medlab Clinical, Sydney 2015, Australia; joyce_zhou@medlab.co (J.Z.); rachel_manuel@medlab.co (R.M.); serena_dalforno@medlab.co (S.D.F.); sean_hall@medlab.co (S.H.); david_rutolo@inmedtech.co (D.R.)

**Keywords:** nanoparticles, NanoCelle, vitamin B_12_, methylcobalamin, cyanocobalamin, liposome, emulsion, tablets

## Abstract

The administration of biological compounds that optimize health benefits is an ever-evolving therapeutic goal. Pharmaceutical and other adjunctive biological compounds have been administered via many different routes in order to produce a systemic pharmacological effect. The article summarizes the findings from an Australian comparative study in adults administered vitamin B_12_ through different oral delivery platforms. A total of 16 subjects (9 males, 7 females) voluntarily partook in a comparative clinical study of five different vitamin B_12_ formulations across a six-month period, completing 474 person-hours of cumulative contribution, that was equivalent to an *n* = 60 participation. A nanoparticle delivered vitamin B_12_ through a NanoCelle platform was observed to be significantly (*p* < 0.05) better absorbed than all other dose equivalent platforms (i.e., tablets, emulsions, or liposomes) from baseline to 1, 3, and 6 h of the study period. The nanoparticle platform delivered vitamin B_12_ demonstrated an enhanced and significant absorption profile as exemplified by rapid systemic detection (i.e., 1 h from baseline) when administered to the oro-buccal mucosa with no reports of any adverse events of toxicity. The nanoparticle formulation of methylcobalamin (1000 µg/dose in 0.3 mL volume) showed bioequivalence only with a chewable-dissolvable tablet that administered a five times higher dose of methylcobalamin (5000 µg) per tablet. This study has demonstrated that an active metabolite embedded in a functional biomaterial (NanoCelle) may constitute a drug delivery method that can better access the circulatory system.

## 1. Introduction

Biological and pharmaceutical compounds can encompass peptides, proteins, antigens, antibodies, nucleic acids, lipids, vitamins and minerals, phytochemicals, and other nutraceuticals (e.g., coenzyme Q_10_, glucosamine) as well as cell therapies [1,2,3]. Delivery to target tissues can include oral, pulmonary, subcutaneous, intravenous, transdermal, and nasal routes. Pharmaceutical agents can be administered via numerous routes and each has advantages and disadvantages [4]. 

The oral route of administration has often been reported to be an important method of administering pharmaceutical or other agents for a systemic effect [5]. The parenteral route has not routinely been used for self-administration of medications or non-pharmaceutical compounds such as nutraceuticals [6]. However, the conventional oral method of administration that is often employed for small molecules presents a difficult barrier for biological compounds. The intestine–liver axis first-pass metabolism can significantly influence drug or nutraceutical metabolism [7]. Additional gastrointestinal limitations also can significantly influence the metabolism and efficacy of a compound. Such limitations include reduced absorption due to the large molecular sizes of biological compounds, a high degree of enzymatic degradation (i.e., by proteolytic enzymes present in the intestinal epithelia and lumen), or a high degree of chemical instability due to the luminal low-pH environment [7,8].

It is widely recognized that the implementation of nanomaterials in biotechnology merges the fields of material science and biology. Nanoparticles provide a particularly useful platform, demonstrating unique properties with potentially wide-ranging therapeutic applications [9]. The advantages of nanotechnology drugs compared to conventional counterparts lie on the basis of particle size. Pharmaceutical/drug products with nano dimensions can be used at a lower concentration and can lead to early onset of bioactivity [10]. Nano drug delivery systems (nano-pharmaceuticals) can be but not limited to variations in applications that include nanocapsules, nanospheres, nanosponges, nanoemulsions, solid lipid nanoparticles, nanovesicular systems (e.g., niosomes, liposomes—the latter exhibiting evidence of toxicity), molecular systems (inclusion complexes), and nanocrystals. Therefore, nano-pharmaceuticals provide enormous potential in drug delivery as carriers for spatial and temporal delivery of bioactive molecules. 

Recent developments in conjunction with nanomedicine for the co-administration of drugs with lipid compounds have been reported to enhance lymphatic transport [11] an example that employs functional biomaterials to enhance drug delivery. Interestingly, in a postprandial state, lipid–drug conjugates, and lipid-based nanoparticles have been widely studied for the delivery of lipophilic drugs via the lymphatic pathway; these are the frameworks advanced for the liposomal delivery of pharmaceutical [12] and non-pharmaceutical compounds [13], utilising functional biomaterials such as fatty acids to form a micelle. Nanotechonology that encapsulates the idea of manipulating matter at the nanometre range has also been limited by the availability of safety data [14] and drawbacks especially with regard to liposomes [15,16]. Notwithstanding though, nanoparticles such as polymeric micelles, liposomes, and conjugated nanoparticles have inspired the drug development industry [17].

The aim of this clinical study with healthy subjects was to compare the absorption profiles over a 6-h period of five different vitamin B_12_ formulations administered via the oral-intestinal tract (e.g., B_12_ tablets), oral mucosa (e.g., liposome B_12_), and oro-buccal site (e.g., nanoparticle B_12_). Currently, there are no such studies that have investigated the absorptive characteristics of a water-soluble compound such as vitamin B_12_ that in humans has a complex process for the gastrointestinal absorption of dietary vitamin B_12_. Briefly, vitamin B_12_ when it is released from food protein is first bound to haptocorrin (salivary vitamin B_12_-binding protein) in the stomach. Proteolysis of haptocorrin–vitamin B_12_ complex by pancreatic proteases follows in the duodenum. The released vitamin B_12_ then goes on to bind to intrinsic factor (IF, gastric vitamin B_12_-binding protein) in the proximal ileum. The IF–vitamin B_12_ complex can enter mucosal cells in the distal ileum by receptor-mediated endocytosis. Bioavailability of dietary vitamin B_12_ is significantly dependent on this gastrointestinal absorption [18] and disease processes can disturb the proper uptake of the vitamin [19]. Moreover, in addition to individuals preferring to avoid intramuscular injections of vitamin B_12_ reports strongly suggest that switching from intramuscular injections to orally delivered formulations of B_12_ significantly lowers costs and benefits the health care system [20]. Therefore, as such this study purports to advance a nanoparticle biomaterial micelle platform complex with a focus of delivering an active ingredient; where the primary focus can be applied to any water soluble or insoluble (e.g., atorvastatin, vitamin D_3_) physiologically active compounds for specific clinical targets.

## 2. Methods

### 2.1. Subjects/Samples/Formulations Administered

Samples of peripheral venous blood were collected via venipuncture from a group of healthy volunteers (*n* = 9 males and *n* = 7 females) using a 19-gauge, 1-inch multi-use needle into a 5 mL red-topped (clot-activating) Vacutainer^®^ (McFarlane Medical, Sydney, Australia) tube. A total of 314 blood samples were collected. None of the participants that were invited to participate in the study had a serious or chronic disease diagnosis on induction, nor were they administering any medications at the time of the study; if they were administering any form of supplements at the time of enrolment they were instructed to cease one week prior to participation and during blood sampling. Participant demographics are presented in Table 1. Written informed consent was obtained from each participant before starting the protocol. All participants adhered to an overnight fast and consented to be repeat participants in the clinical study.

**Figure 1:**
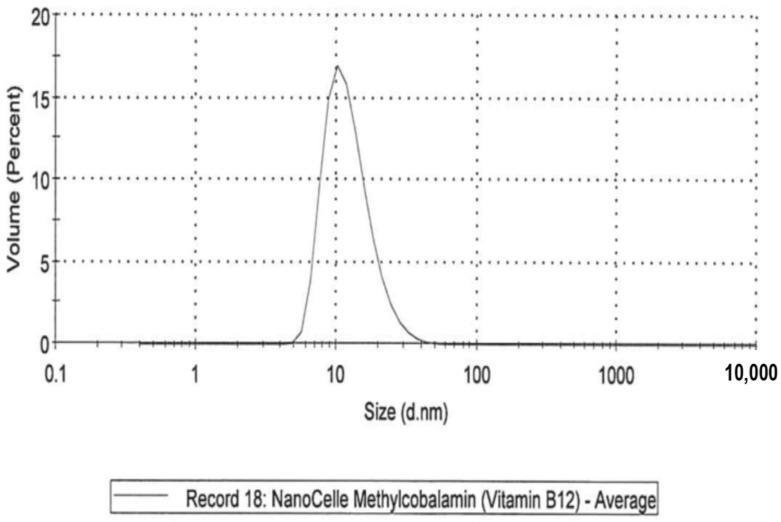
NanoCelle particle size by distribution.

**Figure 2:**
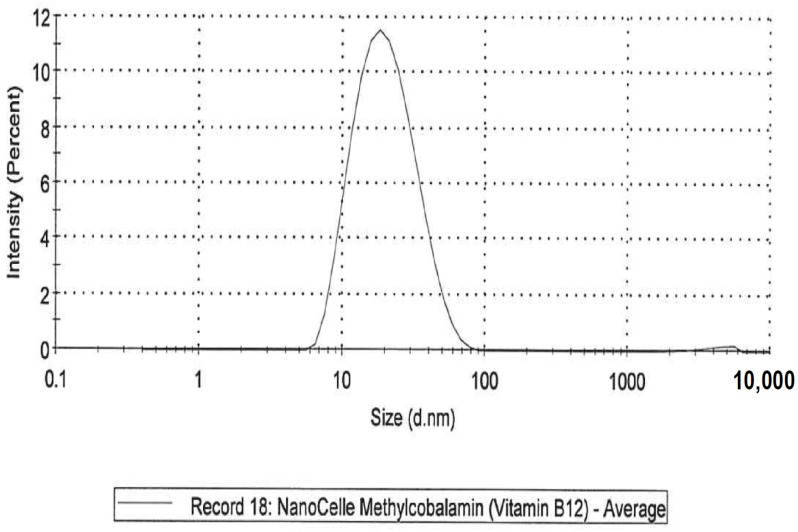
NanoCelle particle size distribution by intensity.

Five formulations were investigated across a six-month period that included:

(i) A NanoCelle (nanoparticle) formulation of B_12_ comprised an oral-buccal spray (Patent: WO2016141069 [21]). This formulation encompasses nano-sized methylcobalamin B_12_ particles (two actuations of the pump delivering 1000 µg/300 µL/dose). The particles consist of an inner hydrophobic core and an outer hydrophilic shell, and has an average particle size of about 20 nm (Figure 1 and Figure 2). Particle analysis was carried out by Malvern Zetasizer from Particle Technology Labs (Chicago, IL, USA).
(ii) An emulsion formulation of B_12_ as cyanocobalamin (two actuations of the pump delivering 1000 µg/340 µL/dose). This formulation was postulated as absorbed through the sublingual mucosa located underneath the tongue by passive diffusion across the membranes.
(iii) A standard tablet formulation of B_12_ as cyanocobalamin (1000 µg/10 tablets/dose) that is absorbed through the gastrointestinal tract.
(iv) A dissolvable (chewable) tablet of B_12_ as methylcobalamin (5000 µg/tablet/dose) that is absorbed through the sublingual mucosa via passive diffusion.
(v) A liposome oral spray formulation of B_12_ as methylcobalamin (two actuations of the pump delivering 1000 µg/300 µL/dose). This formulation provides B_12_ in vesicles constructed of a phospholipid bi-layer, with particle sizes of approximately 100 nm. This nano-sized liposome preparation is posited to assist with the absorption of B_12_ across mucosal membranes.

The National Institute of Integrative Medicine Human Research Ethics Committee (HREC) [EC00436] approved the study. Following HREC approval, the clinical trial received CTN authorization from the TGA (CT-2014-CTN-00485-0-v1) in order to proceed. The clinical trial was registered with the Australian and New Zealand Clinical Trial Registry (ACTRN12616001326482).

### 2.2. Serum Vitamin B_12_ Assay

Blood samples were centrifuged at 3000 x g for 20 min at 4 °C. Serum was separated from blood cells and immediately stored frozen in 1.5 mL micro-centrifuge tubes at −80 °C. Serum samples were then packaged and delivered to a pathology group that was certified by the National Association of Testing Authorities (Australia) for vitamin B_12_ independent assays. The method adopted for assaying serum B_12_ was a chemiluminescence assay per the ADVIA Centaur method [22]. The reported reference range for serum vitamin B_12_ was 301–740 pmoles/L. 

### 2.3. Statistical Analysis

Serum B_12_ values comprise a continuous dataset variable and given that the data were skewed and not normally distributed, results were presented as medians (interquartile ranges). The results were also graphically represented as box plots to demonstrate how disposition of changes occurred in serum levels from baseline through to 1, 3, and 6 h after the administration of the controlled investigational dose of a vitamin B_12_ formulation. Nonparametric tests (Kruskal–Wallis) were used to assess whether significant effects were present from baseline to 6 h for the five different vitamin B_12_ formulations administered. 

## 3. Results

Healthy males (*n* = 9) and females (*n* = 7) in a ratio of approximately 1:1 volunteered to participate in a comparative absorption study of five vitamin B_12_ formulations. Study duration was six months. Demographic variables remained constant throughout the study (Table 1).

**Figure 3:**
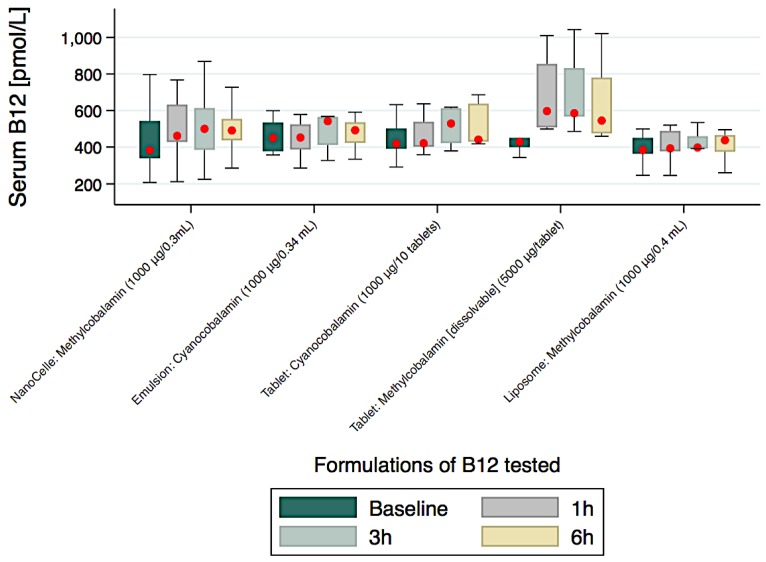
Serum B_12_ disposition from baseline to 6 h for the five formulations tested.

Participants provided a total 314 serum samples for B_12_ assays over the course of the clinical study and the comparisons of levels achieved over a 6-h study period are graphically presented in Figure 3 and numerically with significant trends (medians (IQR)) in Table 2. 

With the exception of the high vitamin B_12_ concentrated (5000 µg/tablet) chewable-dissolvable tablet, the nanoparticle formulation showed the most rapid increase and sustained blood serum concentrations of vitamin B_12_ over the time course of the clinical study. Furthermore, there was a significantly increased serum levels of vitamin B_12_ from baseline to 1 and 3 h for only the nanoparticle delivered platform when compared to all other by equivalent dose formulations administered irrespective of mode of delivery (i.e., emulsion, tablet or liposome) (Table 2). At the 6-h time point, the serum level of B_12_ began to decrease for all formulations. The serum level of the nanoparticle B_12_ formulation showed a 28% increase from baseline at 6-h. The liposome and emulsion formulations showed very low serum levels of B_12_ achieved over the 6-h study period. The liposome formulation was associated with the poorest absorption profile over the study time. The nanoparticle B_12_ formulation was observed to be bioequivalent to a tablet containing five-times higher the B_12_ dose over the 6 h of study (Table 2). 

No adverse events were reported to any of the formulations tested.

## 4. Discussion

The clinical study compared the absorption of vitamin B_12_ by investigating five different delivered formulations and showed that on an equivalent dose basis (1000 µg dose) a nanoparticle (NanoCelle) platform was significantly better at delivering vitamin B_12_ as methylcobalamin than was a tablet, emulsion, or liposome formulation of either methylcobalamin or cyanocobalamin. Furthermore, the nanoparticle B_12_ formulation (1000 µg/dose in 0.3 mL volume) demonstrated bioequivalence with a dissolvable/chewable concentrated per tablet containing five times the dose of B_12_ (i.e., 5000 µg). The innovation of the NanoCelle is in the application of a delivery system that is posited to deliver a nutraceutical or a pharmaceutical directly into the facial lymphatics and hence into the systemic circulation via the oro-buccal mucosa. As such, bypassing intestinal enzymic degradation processes and first pass metabolism of the liver could enhance target tissue delivery of an active compound. 

Oral mucosa formulations for the delivery of supplements such as vitamins, minerals, and active pharmaceutical ingredients include tablets, capsules (hard and soft shelled), lozenges, powders, emulsions, and liquids. In order to benefit from such formulations and ensure optimum absorption of the supplements or active pharmaceutical ingredients, the subject must have a well-functioning gastrointestinal system that ensures adequate absorption via the gastrointestinal tract. It is generally accepted that the dissolution rate of drugs in the intestinal tract affects the absorption rate and the degree to which drugs are absorbed [23]. 

Disease processes can significantly influence the absorption of water-soluble vitamins in the intestines [24]. It is hence biologically plausible to investigate alternative routes of administration of water-soluble compounds such as vitamin B_12_ by employing and exploring the efficacy of nanoparticle technology in oral mucosal delivery systems. 

Research with nanoparticles, and especially liposomes, has been ongoing for more than three decades. However, the development of methods and standard protocols for safety, tolerability, and efficacy testing is still in a developmental phase. This is particularly relevant to liposomes, as this study has shown that liposome delivery of vitamin B_12_ was significantly less efficient than any of the other formulations on dose equivalency. Moreover, of concern was the observation that the serum level of the liposome vitamin B_12_ formulation remained almost constant over the 6-h study period, indicating very low release of the active ingredient and perhaps the systemic and cellular accumulation of the liposomes. 

Accumulation of potentially noxious byproducts promotes the toxicity argument, principally due to the small nanoparticle sizes (or stealth-like characteristics) and the potential for peripheral tissue cellular retention and subsequent toxicity [25]. This potential is detrimental in establishing half-life models for the nanomedicine and a mechanistic understanding of the potential for late stage side effects. It should be appreciated that over the past decade, nanotechnology as nanomedicine has evolved, with a number of pharmaceutical and biotechnology companies undertaking both pharmacokinetic and pharmacodynamic research in efforts to establish toxicology and safety profiles. To this end, we have seen validated research in a handful of drugs, notably liposomal chemotherapeutic agents (e.g., Doxil) where toxicology from the liposomal delivery mechanism was less of an issue due to the presence of the active pharmaceutical ingredient (API).

Of note however, is the scarcity of publications that show liposomal delivery systems associated with various APIs and the reported adverse events that continue to fuel a ‘real’ concern as to their overall efficacy, safety, and toxicity profiles. In this regard, factors of significant concern include [26,27,28,29] liposome preparations that have been described to activate complement component C310; polyethelene glycol that is used in liposomes that play a role in diverse complement activation pathways; liposomes that may trigger the innate immune system response; and liposomes that may also induce immunogenicity [26,27,28,29]. 

It is scientifically implausible to assume that the safety demonstrated through research on one API can be surrogated and translated to other preparations. In reviewing the literature, and the Australian and New Zealand Clinical Trials Register, there is a lack of registrations for liposomal clinical trials on nutraceuticals. It is therefore important to query the safety and efficacy of the use of the liposomal delivery system with any API. 

However, this comparative study has demonstrated that nanoparticle formulations can successfully and safely deliver an API, bypassing the gastrointestinal tract when administered via the oro-bucal site accessing the facial lymphatics, and then passing into the systemic circulation.

## 5. Conclusions

Non-traditional routes of API administration offer an enhanced level of convenience to the patient. These include transportability, reduced cross-contamination issues, and better absorption of the API. Therefore, these therapeutic opportunities offer faster transit times to the circulation and target tissues. Furthermore, because of greater API recovery in serum, less of the API is required for clinical efficacy as compared to standard delivery platforms, which may suffer from loss to first-pass metabolism (e.g., tablets).

Whilst historically nano-delivery systems have had reported safety concerns, improvements in the technology of delivery platforms are putting nanoparticle platforms back into the forefront of clinical practice. Whilst further investigations are warranted, endpoints from this vitamin B_12_ comparative study demonstrated real-world, safe use of a nanoparticle formulation that allowed for fast and safe delivery as evidenced by no adverse events reported.

## Figures and Tables

**Table 1:** Participant demographics.

Demographic Variable	Males (9)	Females (7)
Age (years) mean (SD)	37 (11.8)	31.4 (5.9)
BMI (Kg/m^2^)	27.9 (4.6)	23.6 (2.1)
Systolic BP (mmHg)	132.3 (15.3)	119.3 (4.2)
Diastolic BP (mmHg)	84.8 (8.9)	85.3 (7.6)
Allergies	–	–
Yes	2	2
No	7	5

**Table 2:** Serum B_12_ levels achieved from five different formulations over 6 h.

Collection Times	Formulations				
	Nanocelle Ω 1000 µg/0.3 mL	Emulsion 1000 µg/0.3 mL	Tablet 1000 µg/Tablet	Chewable 5000 µg/Tablet ^⊥^	Liposome 1000 µg/0.3 mL
Serum B_12_ Reported as Median (IQR) pmoles/L					
Baseline	383 (204)	450 (157)	419 (110)	429 (50)	385 (86)
1 h	462 (205) *	453 (138)	421 (137)	597 (327) *	393 (112)
%↑baseline-to-1 h	21% (*p* < 0.05)	1%	0.5%	39%	2%
3 h	500 (229) *	542 (154)	529 (191)	586 (265) *	398 (67)
6 h	491 (118) *	493 (114)	441 (208) *	545 (305) *	438 (92) *
%↑baseline-to-6 h	28% (*p* < 0.05)	10%	5%	27%	14%

Ω nanoparticle formulation; * Serum levels of B_12_ statistically significantly increased differences from baseline; ⊥ Dissolvable/Chewable Tablet with 5 times higher the B_12_ content.
